# Heterogeneity in Prevalence, Incidence, and Clearance of Anal Human Papillomavirus Among HIV-Negative and HIV-Positive Men Who Have Sex with Men in China: An Observational Cohort Study

**DOI:** 10.3390/vaccines13111144

**Published:** 2025-11-07

**Authors:** Tian Tian, Zhen Lu, Jingjing He, Leiwen Fu, Wenhui Yu, Zewen Zhang, Zhen Chen, Huachun Zou, Jianghong Dai

**Affiliations:** 1 School of Public Health, Xinjiang Medical University, Urumqi 835000, China; tiantian@xjmu.edu.cn (T.T.); jinghe@stu.xjmu.edu.cn (J.H.); wenhuiyu@stu.xjmu.edu.cn (W.Y.);; 2 School of Public Health (Shenzhen), Sun Yat-sen University, Shenzhen 518000, China; luzh29@mail2.sysu.edu.cn; 3 Beijing Chest Hospital, Beijing Tuberculosis and Thoracic Tumor Research Institute, Capital Medical University, Beijing 101149, China; 4 School of Public Health, Fudan University, Shanghai 200032, China; 5 School of Public Health, Southwest Medical University, Luzhou 646000, China; 6 Kirby Institute, University of New South Wales, Sydney NSW 2052, Australia; 7 Key Laboratory of Special Environment and Health Research in Xinjiang, Urumqi 835000, China

**Keywords:** men who have sex with men, HPV, HIV, natural history

## Abstract

Background: Men who have sex with men (MSM) are at high risk for anal human papillomavirus (HPV) infection, with HIV-positive MSM bearing the highest disease burden. Longitudinal data on anal HPV infection among HIV-negative and HIV-positive MSM are limited. We assessed and compared the prevalence, incidence, and clearance of anal HPV infection among HIV-negative and HIV-positive MSM in Xinjiang, China. Methods: Sexually active HIV-positive and HIV-negative MSM aged 18 years and older have been enrolled in an ongoing observational cohort study of HPV since 1 September 2016, in Xinjiang, China. Participants were followed up on every 6 months with anal HPV testing and questionnaires regarding sexual behaviors. We compared HPV prevalence, incidence, and clearance between HIV-positive and HIV-negative MSM. Prevalence ratios (PRs), incidence rate ratios (IRRs), and clearance rate ratios (CRRs) for HIV-negative and HIV-positive MSM were calculated. Results: A total of 1425 MSM, including 131 HIV-positive and 1294 HIV-negative individuals, with a median age of 29 years (interquartile range [IQR]: 24 to 36), were included in our analysis. Compared with HIV-negative MSM, HIV-positive MSM demonstrated significantly higher prevalence across both individual and grouped HPV genotypes. Specifically, the prevalence of grouped HPV genotypes (any, high-risk, low-risk, 9v, 4v, HPV16/18, and HPV 6/11) was consistently elevated in HIV-positive individuals. PRs for individual HPV types 31, 45, 34, 44, 53, and 81 were 2.47 (95% CI: 1.16–5.25), 2.47 (1.10–5.54), 4.94 (1.25–19.52), 3.29 (1.08–10.06), 2.02 (1.01–4.04), and 2.66 (1.18–6.01), respectively. Furthermore, the incidence of most individual HPV genotypes were higher, while the clearance rates were lower among HIV-positive MSM. Specifically, IRRs for HPV types 31, 33, 45, 55, and 66 were 2.12 (1.19–3.75), 2.19 (1.24–3.90), 2.32 (1.17–4.59), 3.02 (1.15–7.93), and 2.44 (1.18–5.05), respectively. CRRs for HPV types 51 and 58 were 0.33 (0.21–0.52) and 0.60 (0.45–0.79), respectively. Conclusions: HPV prevalence, incidence, and clearance of anal HPV exhibited heterogeneity between HIV-positive and HIV-negative MSM. HPV vaccination and condom promotion programs should be recommended for HIV-positive MSM to mitigate the burden of HPV infection in this vulnerable population.

## 1. Background

The Papillomaviridae family comprises over 450 distinct human papillomavirus (HPV) types that primarily infect basal epithelial cells [1,2]. Based on their association with cancer development, HPV can be categorized into low-risk and high-risk types [3]. Low-risk HPV types, such as HPV 6 and HPV 11, can cause benign conditions such as genital warts. In addition to being a necessary cause of all cervical cancers, high-risk HPV is responsible for approximately 88% of anal cancers worldwide [4].

HPV vaccination, coupled with cervical screening, offers the most effective protection against HPV-related cervical cancer. However, prevention efforts have been less successful in addressing HPV-related non-cervical cancers. The incidence of anal cancer has been increasing significantly over the past decades in most countries [5,6]. Notably, men who have sex with men (MSM) are disproportionately affected by HPV-related anal cancer. A recent meta-analysis indicates that the incidence of anal cancer among MSM is approximately 20 cases per 100,000 person years [7].

HIV status has also been recognized as an important determinant of anal cancer in men. HIV, primarily through its immunosuppressive effects, may facilitate the acquisition and persistence of HPV among MSM, thereby exacerbating the risk of anal cancer among HIV-positive MSM [8]. The incidence of anal cancer is estimated at approximately 85 per 100,000 person years for HIV-positive MSM [7].

Potential strategies for anal cancer prevention can be divided into primary prevention (e.g., HPV vaccination) and secondary prevention (e.g., screening of high-risk populations), which aim to detect and manage high-grade squamous intraepithelial lesions. Epidemiological data on anal HPV infection in target populations can guide anal cancer prevention programs for MSM and predict their potential impact. Extensive data on anal HPV prevalence are available for both HIV-negative and HIV-positive MSM, providing a solid basis for robust comparative analyses. Across all individual and grouped HPV types, the prevalence of anal HPV is consistently higher among HIV-positive MSM compared to HIV-negative MSM and HIV-positive heterosexual men.

Longitudinal data on the differences in the incidence and clearance of anal HPV between HIV-positive MSM and HIV-negative MSM remain limited yet are critical for the development of precise strategies of anal cancer prevention in this population. This study aims to investigate the incidence and clearance persistence of anal HPV infection in a longitudinal cohort of HIV-positive and HIV-negative MSM in Xinjiang, China.

## 2. Methods

### 2.1. Overview

This research utilized data from an ongoing prospective cohort investigating HPV dynamics among MSM in Xinjiang, China. The cohort was established to characterize HPV infection patterns and inform targeted prevention strategies for this key population.

### 2.2. Study Population

Details of the methods used in the HPV cohort study have been published elsewhere [9]. This prospective cohort study recruited participants from September 2016 to April 2025. Briefly, recruitment commenced on 1 September 2016 through Xinjiang Dream Health Service Center, a community-based Non-Governmental Organizations (NGO) serving sexual minority populations. Participants were eligible if they were 18 years and older and reported engaging in male–male anal/oral intercourse within the preceding six months.

### 2.3. Data Sources and Measurement

Standardized specimen collection protocols for this cohort are described in our foundational publication [10]. This prospective cohort study was conducted from September 2016 to April 2025 to investigate the heterogeneity in the prevalence, incidence, and clearance of anal HPV among HIV-negative and HIV-positive MSM in China. Participants provided anal swab specimens at baseline and subsequent six-month intervals. NGO staff, trained by clinical specialists, performed specimen collection following standardized operating procedures.

All specimens underwent centralized processing at Xinjiang using the Hybribio 37 HPV GenoArray Diagnostic Kit (Hybribio Biotech Co., Ltd., Jieyang, China), which identifies 37 HPV genotypes through HybriMax analysis. This testing kit can detect 37 common types of HPV, including 22 high-risk genotypes (16, 18, 26, 31, 33, 34, 35, 39, 45, 51, 52, 53, 56, 58, 59, 66, 67, 68, 69, 70, 73, and 82) and 15 low-risk genotypes (6, 11, 40, 42, 43, 44, 54, 55, 57, 61, 71, 72, 81, 83, and 84). Samples lacking detectable β-globin and HPV DNA were excluded as invalid. HIV testing followed China’s national guidelines: Initial screening with Alere HIV 1/2 Combo rapid test (Abbott Laboratories. Chicago, IL, USA), with reactive results confirmed by HIV 1/2 STAT-PAK (Chembio Diagnostics, Inc. Hauppauge, NY, USA). Pre- and post-test counseling accompanied all HIV testing procedures. Participants completed structured questionnaires at each visit capturing: demographic characteristics, sexual behavior history (debut age, partner numbers, sexual positioning roles), condom usage patterns, substance use during sex, commercial sex exposure, circumcision status, and self-reported STI history within six months. Each visit included RMB 40 compensation.

### 2.4. Outcomes

The primary endpoints assessed in this study included the prevalence, incidence, and clearance of anal HPV infection. At baseline, we evaluated the prevalence of any of the 37 HPV genotypes; any high-risk types (HPV 16, 18, 26, 31, 33, 34, 35, 39, 45, 51, 52, 53, 56, 58, 59, 66, 67, 68, 69, 70, 73, 82); any low-risk types (HPV 6, 11, 40, 42, 43, 44, 54, 55, 57, 61, 71, 72, 81, 83, 84); genotypes covered by the 9-valent HPV vaccine (6, 11, 16, 18, 31, 33, 45, 52, 58); those in the quadrivalent vaccine (6, 11, 16, 18); co-infection with HPV 6/11; co-infection with HPV 16/18; as well as each individual genotype. A genotype was considered prevalent if it was detectable at baseline. Participants acquiring HPV infection during follow-up were classified as incident cases. Specifically, incident infection was defined as the first detection of a specific HPV genotype after an initial negative result (i.e., transition from 0 to 1). The time at risk for incidence calculation started from the first genotype-specific negative visit and ended at the date of first positive detection. Clearance was defined as a subsequent negative result following a prior positive detection of the same genotype (transition from 1 to 0). Participants became at risk for clearance from the first positive test and exited either at the time of clearance or at the last positive visit if no clearance occurred. Persistence of HPV infection was defined as at least two consecutive positive results for the same genotype, while persistent infection of any HPV type required at least two successive positive tests for one or more genotypes.

### 2.5. Statistical Analysis

Descriptive statistics were used to characterize demographic and sexual behavior profiles across all participants. Continuous variables were expressed as medians with interquartile ranges (IQRs), and categorical variables were summarized as frequencies and proportions. For group comparisons of non-normally distributed continuous variables (e.g., age at first anal intercourse), the Mann–Whitney U test was employed. Differences in baseline characteristics and sexual behaviors between HIV-positive and HIV-negative MSM were examined using the Wilcoxon rank-sum test for continuous variables and either the Chi-square test or Fisher’s exact test for categorical variables, as appropriate.

Prevalence ratios (PRs) and 95% confidence intervals (CIs) were calculated to compare HPV prevalence between HIV-positive and HIV-negative individuals. Incidence rates (IRs) and clearance rates (CRs) were reported per 1000 person-months, determined by dividing the number of incident or cleared infections by total person-time at risk. Only a single incident or clearance event per genotype was considered for each individual, and each event was treated as independent. Confidence intervals for IRs and CRs were derived using exact Poisson methods. Incidence rate ratios (IRRs) and clearance rate ratios (CRRs), along with their corresponding 95% CIs, were estimated using Poisson regression models to assess differences between HIV-positive and HIV-negative MSM. All *p*-values were two-sided, with a significance level set at α = 0.05. Statistical analyses were conducted using R version 4.0.4 (R Foundation for Statistical Computing, Vienna, Austria).

## 3. Results

### 3.1. Description of Study Population

From September 2016 to April 2025, a total of 1469 MSM were present in the HPV cohort, 1425 had adequate β-globin for HPV genotype testing and were included in this analysis. The median age of the participants upon entry into the study was 29 years (IQR: 24–36). Among them, 719 (50.46%) had bachelore’s degree or above. 1110 (77.89%) self-identified as gay. At baseline, 1155 (81.05%) participants reported engaging in anal sex within the previous six months.

Of the 1425 participants, 131 (9.2%) were HIV-positive. Most of the demographic characteristics and sexual behaviors did not significantly differ between HIV-positive and HIV-negative participants (Table 1). However, significant disparities in education level and monthly income were observed between HIV-positive and HIV-negative participants. The HIV-positive group exhibited lower proportions of individuals with bachelor’s degree and above attainment (35.1% vs. 52.0%, *p* < 0.001). Additionally, 60.3% of HIV-positive participants reported monthly incomes between 1001–5000 yuan, significantly exceeding the 47.1% proportion in the HIV-negative group (*p* = 0.008). HIV-positive participants demonstrated significantly earlier median age at first anal intercourse (20.0 [IQR 18.0–23.0] vs. 20.0 [18.0–22.0] years, *p* = 0.016) and higher substance use rates (40.5% vs. 26.1%, *p* < 0.001). Commercial sex engagement was less prevalent among HIV-positive individuals (1.5% vs. 4.4%, *p* = 0.049). No significant differences were observed in other sexual behaviors.

### 3.2. HPV Prevalence

**Figure 1:**
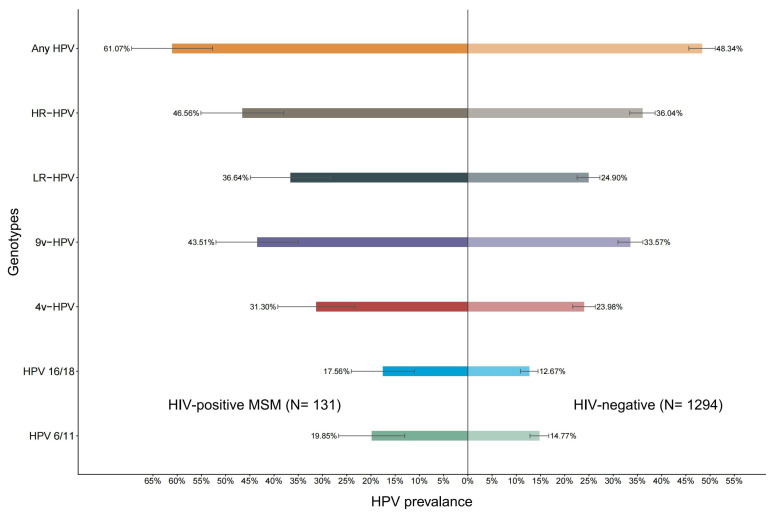
The prevalence of grouped HPV genotypes among HIV-positive and HIV-negative MSM. HPV, human papillomavirus; High-risk HPV: HPV genotypes 16, 18, 31, 33, 35, 39, 45, 51, 52, 56, 58, 59, and 68; Low-risk HPV: HPV genotypes 6, 11, 26, 34, 40, 42, 43, 44, 53, 54, 55, 57, 61, 66, 67, 69, 70, 71, 72, 73, 81, 82, 83, 84; 9vHPV: HPV genotypes 6, 11, 16, 18, 31, 33, 45, 52, 58; 4vHPV: HPV genotypes 6, 11, 16, 18; HPV 6/11: HPV genotype 6 and/or 11; HPV 16/18: HPV genotype 16 and/or 18.

Figure 1 shows the prevalence of grouped HPV genotypes of different groups. For HIV-positive individuals, the prevalence of any, high-risk, low-risk, 9v, 4v, HPV16/18, and HPV 6/11 genotypes was 61.07 (95% CI: 53.26–70.02), 46.56 (95% CI: 38.02–55.11), 36.64 (95% CI: 28.39–44.89), 43.51 (95% CI: 35.80–52.89), 31.30 (95% CI: 24.28–40.34), 17.56 (95% CI: 12.11–25.45), and 19.85 (95% CI: 14.07–28.00), respectively. The prevalence of grouped HPV genotypes, including any, high-risk, low-risk, 9v, 4v, HPV16/18, and HPV 6/11 genotypes were higher among HIV-positive individuals in comparison to HIV-negative individuals.

The prevalence of individual HPV genotypes of HIV-positive individuals and HIV-negative individuals was presented in Table 2. For HIV-positive individuals, the most prevalent high-risk HPV genotypes were HPV 58 and 16, with a prevalence of 12.98% (95% CI: 8.33–20.22) and 12.21% (95% CI: 7.72–19.33), respectively. While the most prevalent low risk HPV genotypes among HIV-positive individuals were HPV 6 and 11, with a prevalence of 13.74% (95% CI: 8.95–21.10) and 9.16% (95% CI: 5.34–15.71), respectively. For HIV-negative individuals, the most prevalent high-risk HPV genotypes were HPV 16 and 39, with a prevalence of 9.27% (95% CI: 7.82–11.00) and 6.42% (95% CI: 5.21–7.90), respectively. While the most prevalent low risk HPV genotypes among HIV-negative individuals were HPV 6 and 11, with a prevalence of 9.13% (95% CI: 7.68–10.84) and 6.34% (95% CI: 5.14–7.81), respectively.

The prevalence of the majority of the 37 individual HPV genotypes was higher among HIV-positive individuals in comparison to HIV-negative individuals. Specifically, among high-risk HPV genotypes, the prevalence of HPV 31, 45, and 58 was significantly higher in HIV-positive individuals compared to HIV-negative individuals, with PRs of 2.47 (95% CI: 1.16–5.25), 2.47 (95% CI: 1.10–5.54), and 2.75 (95% CI: 1.66–4.57), respectively. For low-risk HPV genotypes, the prevalence of HPV 34, 44, 53, 54, 66, and 81 genotypes was significantly higher in HIV-positive individuals compared to HIV-negative individuals, with PRs of 4.94 (95% CI: 1.25–19.52), 3.29 (95% CI: 1.08–10.06), 2.02 (95% CI: 1.01–4.04), 3.66 (95% CI: 1.81–7.39), 4.70 (95% CI: 2.26–9.77), and 2.66 (95% CI: 1.18–6.01), respectively.

### 3.3. HPV Incidence

Individual HPV genotypes IRs of HIV-positive individuals and HIV-negative individuals were presented in Table 3. Among HIV-positive individuals, the high-risk HPV genotypes with the highest incidence rates were HPV 16 and 51, with incidence rates of 25.00 (95% CI: 16.94–36.90) and 15.58 (95% CI: 9.27–26.21) per 1000 person-months, respectively. The low-risk HPV genotypes with the highest incidence rates among HIV-positive individuals were HPV 6 and 84, with incidence rates of 22.67 (95% CI: 14.92–34.43) and 14.81 (95% CI: 8.79–24.97) per 1000 person-months, respectively. Among HIV-negative individuals, the high-risk HPV genotypes with the highest incidence rates were HPV 16 and 52, with incidence rates of 18.54 (95% CI: 16.11–21.35) and 13.86 (95% CI: 11.74–16.36) per 1000 person-months, respectively. The low-risk HPV genotypes with the highest incidence rates among HIV-negative individuals were HPV 6 and 11, with incidence rates of 17.76 (95% CI: 15.37–20.51) and 13.79 (95% CI: 11.68–16.29) per 1000 person-months, respectively.

The IRs of 37 individual HPV genotypes among HIV-positive individuals were found to be slightly higher than HIV-negative MSM. For high-risk HPV genotypes, the incidence of HPV 31, 33, and 45 was significantly higher among HIV-positive individuals in comparison to HIV-negative individuals, with an IRR of 2.12 (95% CI: 1.19–3.75), 2.19 (95% CI: 1.24–3.90), and 2.32 (95% CI: 1.17–4.59), respectively. For low-risk HPV genotypes, the incidence of HPV 55, 66, and 84 was significantly higher among HIV-positive individuals in comparison to HIV-negative individuals, with an IRR of 3.02 (95% CI: 1.15–7.93), 2.44 (95%CI: 1.18–5.05), and 3.50 (1.90–6.45), respectively.

### 3.4. HPV Clearance

Individual HPV genotypes CRs of HIV-positive individuals and HIV-negative individuals were presented in Table 4. Among HIV-positive individuals, the high-risk HPV genotypes with the lowest clearance rates were HPV 51 and 68, with clearance rates of 30.77 (95% CI: 19.91–47.55) and 50.00 (18.44–135.6) per 1000 person-months, respectively. The low-risk HPV with the lowest clearance rates were HPV 6 and 53, with clearance rates of 63.64 (95% CI: 47.68–84.93) and 70.00 (95% CI: 47.76–102.6) per 1000 person-months, respectively. Among HIV-negative individuals, the high-risk HPV genotypes with the lowest clearance rates were HPV 56 and 16, with clearance rates of 82.14 (95% CI: 65.63–102.8) and 83.98 (95% CI: 76.80–91.83) per 1000 person-months, respectively. The low-risk HPV with the lowest clearance rates were HPV 6 and 11, with clearance rates of 85.42 (95% CI: 78.51–92.93) and 86.86 (95% CI: 79.38–95.05) per 1000 person-months, respectively.

The CRs of the majority of the 37 individual HPV genotypes were comparable among HIV-positive individuals and HIV-negative individuals. Nevertheless, the CRs of HPV 51 and 58 genotypes were notably lower in HPV-positive individuals in comparison to HIV-negative individuals, with a CRR of 0.33 (95% CI: 0.21–0.52) and 0.60 (95% CI: 0.45–0.79), respectively.

## 4. Discussion

This longitudinal study followed 1425 MSM from September 2016 to April 2025, systematically evaluating the epidemiological characteristics of anal HPV infection among HIV-positive and HIV-negative MSM in China, which was one of the few cohort evaluations assessing the prevalence, incidence, and clearance among HIV-positive and HIV-negative MSM. The study findings reveal that HIV-positive MSM demonstrate higher prevalence and incidence for most HPV types compared to HIV-negative MSM, alongside reduced HPV clearance rates; however, these differences reached statistical significance for only a limited number of specific HPV genotypes. The findings provided critical evidence for HPV prevention and control among MSM.

This study delineates distinct socioeconomic profiles between HIV-positive and HIV-negative MSM populations. Notably, educational disparities emerged as a prominent differentiating factor, with over 50.0% of HIV-negative participants obtaining bachelor’s degrees or higher, contrasting sharply with 35.1% attainment in the HIV-positive MSM. This educational gradient has been corroborated by both domestic and international investigations, including parallel findings from South African cohorts demonstrating comparable patterns of educational disadvantage among HIV-positive MSM [11,12,13,14]. Lower educational attainment is associated with weaker HIV prevention awareness, reduced protective behaviors, and insufficient understanding of transmission risks, collectively elevating susceptibility to HIV infection [14]. Similarly, the increased risk of HPV infection also appears attributable to analogous deficiencies in risk perception and preventive health knowledge caused by low educational attainment. Moreover, compared to HIV-negative MSM, HIV-positive MSM exhibited younger age at first sexual intercourse and higher rates of substance use. Studies have shown that these factors may represent significant risk factors for HPV and other STIs, including HIV. Research indicates that earlier sexual debut and substance use is associated with increased probability of engagement in unprotected sexual behaviors, which may lead to elevated infection risks [15,16].

In this study, we found that HIV-positive MSM exhibited higher HPV prevalence compared to HIV-negative MSM [17,18,19]. The results aligned with global data demonstrating elevated anal HPV prevalence among HIV-positive MSM [20,21]. Specifically, we found that HPV 58 was the most prevalent type, the prevalence was significantly higher among HIV-positive MSM than HIV-negative MSM. Combined with high prevalence of HPV 6 and 11 in this population, this limits the effectiveness of the 2v or 4v vaccine against HPV-related diseases in this group. Thus, the 9v HPV vaccine represents a more optimal choice for HIV-positive MSM [22]. Based on long-term follow-up, this study innovatively documented the differential HPV incidence between HIV-positive and HIV-negative MSM. The results indicate that the incidence of HPV was significantly higher in HIV-positive individuals than in HIV-negative MSM. The higher incidence of HPV among HIV-positive individuals may be attributed to lower educational attainment and high-risk sexual behaviors (including substance use). Another important mechanism was the immunodeficiency induced by HIV, characterized by depletion of CD4+ T-cells and compromised immune function, impairing the host’s capacity to mount an effective immune response against new HPV infections.

Moreover, HIV-positive MSM exhibited markedly reduced clearance for multiple high-risk HPV types, particularly HPV 51 and HPV 58, compared with HIV-negative participants. This suggests a heightened risk of persistent infection in this immunocompromised population—a pattern consistent with previous longitudinal studies in similar cohorts [18,23]. The prolonged persistence of these genotypes underscores the role of HIV-associated immunosuppression in impeding HPV clearance. These findings indicate that the elevated HPV susceptibility in HIV-positive MSM necessitates integrating HPV vaccination and systematic screening into HIV care to reduce their disproportionate HPV-related disease burden. Notably, although previous studies had consistently reported HPV 16 showing the lowest clearance rates in MSM [23,24], our findings identified HPV 51 and HPV 58 as having the poorest clearance among HIV-positive MSM. Combined with the high prevalence, incidence, and low clearance of 2v and 4v HPV vaccine targeted types among HIV-positive MSM, our results suggest that 2v and 4v HPV vaccines may not adequately address the oncogenic HPV burden in this population. The implementation of HPV vaccines that covers more types or tailored prevention strategies is therefore imperative. However, currently, there is not enough data on comparative effectiveness or on the evaluation of harms and benefits of prevention strategies to recommend a preferred option. Further, longitudinal studies evaluating different screening approaches are lacking, making it difficult to recommend evidence-based intervals for screening and management.

This study had several limitations. First, The use of convenience sampling, predominantly involving college-educated participants from Xinjiang, China, limits the generalizability of the findings to broader MSM populations. Second, although we attempted to minimize recall bias and social desirability bias by using computer-assisted self-interview methods, self-reported sexual behaviors may still be subject to these biases. Third, the lack of data on HPV vaccination status may have confounded estimates of anal HPV prevalence and incidence. However, China did not approve the HPV 9-valent vaccine for males until 15 April 2025, so the majority of the MSM should not have been vaccinated. Additionally, the use of baseline values for time-varying exposures such as smoking and alcohol could introduce measurement bias. Another limitation is the imbalance in the sample size between the HIV-positive and HIV-negative groups, which could influence both the significance and generalizability of the results for this subgroup. Therefore, future studies with more balanced designs are needed to confirm these observations. Some estimates in the HIV-positive subgroup also showed wide confidence intervals due to limited sample size, warranting cautious interpretation. Finally, the inability to monitor individuals who declined participation may have resulted in a sample predominantly composed of MSM with heightened interest in HPV. This selection bias could lead to an overestimation of HPV prevalence in the study cohort, potentially over-representing individuals with higher risk factors for HPV, thus impacting prevalence rates in both groups. However, as study mature, this innovative long-term cohort study on the natural history of HPV infection among HIV-positive and HIV-negative MSM will provide the basis for risk-based HPV screening and management recommendations for this at-risk population.

## 5. Conclusions

Our study highlights the disproportionate burden of anal HPV among HIV-positive MSM in China, marked by high prevalence, high incidence, and impaired clearance. Strengthening HPV vaccination coverage, expanding screening programs, implementing comprehensive educational campaigns that emphasize consistent and correct condom use, and integrating HPV prevention into HIV care are critical to reducing anal cancer disparities in this high-risk population.

## Figures and Tables

**Table 1:** Baseline demographics and behavioral characteristics among participants in this study.

Characteristics	HIV-Negative (N = 1294)	HIV-Positive (N = 131)	*p* Value	Total (N = 1425)
Age (years) *	29.00 (24.00, 35.00)	28.00 (23.00, 36.75)	0.209	29.00 (24.00, 36.00)
Locality			0.195	
Local resident	630 (48.69)	56 (42.75)		686 (48.14)
Non-local resident	664 (51.31)	75 (57.25)		739 (51.86)
Ethnicity			0.395	
Han	1112 (85.94)	109 (83.21)		1221 (85.68)
Non-han	182 (14.06)	22 (16.79)		204 (14.32)
Educational level			<0.001	
High school or below	262 (20.25)	43 (32.82)		305 (21.40)
Some college	359 (27.74)	42 (32.06)		401 (28.14)
Bachelor’s degree and above	673 (52.01)	46 (35.11)		719 (50.46)
Employment			0.815	
Employed	1046 (80.83)	107 (81.68)		1153 (80.91)
Unemployed	248 (19.17)	24 (18.32)		272 (19.09)
Salary (yuan/month)			0.008	
≤1000	161 (12.44)	17 (12.98)		178 (12.49)
1001~5000	610 (47.14)	79 (60.31)		689 (48.35)
5001~10,000	419 (32.38)	32 (24.43)		451 (31.65)
≥10,001	104 (8.04)	3 (2.29)		107 (7.51)
Age at first anal intercourse (years) ^#^	20.00 (18.00, 23.00)	20.00 (18.00, 22.00)	0.016	20.00 (18.00, 23.00)
Sexual orientation			0.188	
Gay	1002 (77.43)	108 (82.44)		1110 (77.89)
Bisexual or other	292 (22.57)	23 (17.56)		315 (22.11)
Sexual partner			0.592	
Men only	749 (57.88)	79 (60.31)		828 (58.11)
Both men and women	545 (42.12)	52 (39.69)		597 (41.89)
Sexual partner during last year			0.504	
Men only	1056 (81.61)	110 (83.97)		1166 (81.82)
Both men and women	238 (18.39)	21 (16.03)		259 (18.18)
Anal sex during last six months			0.067	
Yes	1041 (80.45)	114 (87.02)		1155 (81.05)
No	253 (19.55)	17 (12.98)		270 (18.95)
Predominant role in anal sex			0.402	
Mainly insertive	853 (65.92)	94 (71.76)		947 (66.46)
Mainly receptive	247 (19.09)	21 (16.03)		268 (18.81)
Insertive and receptive	194 (14.99)	16 (12.21)		210 (14.74)
Homosexual partner number during last six months ^&^	2.00 (1.00, 3.00)	2.00 (1.00, 3.50)	0.013	2.00 (1.00, 3.00)
Condom use			0.139	
Yes	782 (60.43)	82 (62.60)		864 (60.63)
No	259 (20.02)	32 (24.43)		291 (20.42)
No anal sex during last six months	253 (19.55)	17 (12.98)		270 (18.95)
Frequence of condom use during last six months			0.185	
Always	588 (45.44)	58 (44.27)		646 (45.33)
Sometimes	186 (14.37)	24 (18.32)		210 (14.74)
Never	267 (20.63)	32 (24.43)		299 (20.98)
No anal sex during last six months	253 (19.55)	17 (12.98)		270 (18.95)
Commercial sex			0.049	
No	1001 (77.36)	113 (86.26)		1114 (78.18)
Yes	57 (4.40)	2 (1.53)		59 (4.14)
Unknown	236 (18.24)	16 (12.21)		252 (17.68)
Heterosexual sex during last six months			0.797	
No	1146 (88.56)	117 (89.31)		1263 (88.63)
Yes	148 (11.44)	14 (10.69)		162 (11.37)
Substance			<0.001	
No	956 (73.88)	78 (59.54)		1034 (72.56)
Yes	338 (26.12)	53 (40.46)		391 (27.44)
VCT			0.914	
Yes	1131 (87.40)	114 (87.02)		1245 (87.37)
No	85 (6.57)	8 (6.11)		93 (6.53)
Unknown	78 (6.03)	9 (6.87)		87 (6.11)
Circumcision			0.602	
No	782 (60.43)	85 (64.89)		867 (60.84)
Yes	487 (37.64)	44 (33.59)		531 (37.26)
Unknown	25 (1.93)	2 (1.53)		27 (1.89)
Tobacco			0.092	
Never	622 (48.07)	56 (42.75)		678 (47.58)
Sometimes	295 (22.80)	41 (31.30)		336 (23.58)
Everyday	377 (29.13)	34 (25.95)		411 (28.84)
Alcohol			0.135	
Never	279 (21.56)	28 (21.37)		307 (21.54)
Sometimes	977 (75.50)	103 (78.63)		1080 (75.79)
Everyday	38 (2.94)	0 (0.00)		38 (2.67)

Notes: * 6 missing values for age; ^#^ 10 missing values for age at first anal intercourse; ^&^ VCT, voluntary counseling and testing.

**Table 2:** Baseline prevalence of the individual HPV genotypes among HIV-positive and HIV-negative MSM.

HPV Genotypes	HIV-Positive (N = 131)	HIV-Negative (N = 1294)	Risk Ratio (HIV-Positive vs. HIV-Negative)	*p* Value
HPV16	12.21 (7.72–19.33)	9.27 (7.82–11.00)	1.32 (0.81–2.15)	0.270
HPV18	6.11 (3.12–11.95)	4.25 (3.28–5.50)	1.44 (0.70–2.95)	0.324
HPV31	6.11 (3.12–11.95)	2.47 (1.76–3.48)	2.47 (1.16–5.25)	0.019
HPV33	5.34 (2.60–10.99)	3.40 (2.54–4.55)	1.57 (0.72–3.42)	0.254
HPV45	5.34 (2.60–10.99)	2.16 (1.50–3.12)	2.47 (1.10–5.54)	0.028
HPV52	3.82 (1.62–9.02)	4.33 (3.35–5.59)	0.88 (0.36–2.16)	0.784
HPV58	12.98 (8.33–20.22)	4.71 (3.69–6.02)	2.75 (1.66–4.57)	<0.001
HPV6	13.74 (8.95–21.10)	9.13 (7.68–10.84)	1.51 (0.95–2.39)	0.083
HPV11	9.16 (5.34–15.71)	6.34 (5.14–7.81)	1.45 (0.81–2.58)	0.212

HPV, human papillomavirus.

**Table 3:** Incidence of the individual HPV genotypes among HIV-positive and HIV-negative MSM.

HPV Genotypes	HIV-Positive (N = 131)	HIV-Negative (N = 1294)	Rate Ratio (HIV-Positive vs. HIV-Negative)	*p* Value
HPV16	25.00 (16.94–36.90)	18.54 (16.11–21.35)	1.35 (0.89–2.04)	0.157
HPV18	6.33 (2.71–14.78)	11.03 (9.14–13.33)	0.57 (0.24–1.37)	0.210
HPV31	15.19 (9.02–25.58)	7.18 (5.66–9.10)	2.12 (1.19–3.75)	0.010
HPV33	15.38 (9.14–25.89)	7.01 (5.50–8.93)	2.19 (1.24–3.90)	0.007
HPV45	11.11 (6.00–20.57)	4.79 (3.57–6.44)	2.32 (1.17–4.59)	0.016
HPV52	10.13 (5.25–19.53)	13.86 (11.74–16.36)	0.73 (0.37–1.44)	0.364
HPV58	10.81 (5.62–20.80)	10.71 (8.84–12.96)	1.01 (0.51–2.00)	0.978
HPV6	22.67 (14.92–34.43)	17.76 (15.37–20.51)	1.28 (0.82–1.99)	0.279
HPV11	13.92 (8.05–24.09)	13.79 (11.68–16.29)	1.01 (0.57–1.79)	0.974

HPV, human papillomavirus.

**Table 4:** Clearance of the individual HPV genotypes among HIV-positive and HIV-negative MSM.

HPV Genotypes	HIV-Positive (N = 131)	HIV-Negative (N = 1294)	Rate Ratio (HIV-Positive vs. HIV-Negative)	*p* Value
HPV16	74.07 (57.90–94.77)	83.98 (76.80–91.83)	0.88 (0.68–1.15)	0.348
HPV18	85.71 (63.88–115.0)	93.58 (87.14–100.5)	0.92 (0.68–1.24)	0.570
HPV31	76.92 (59.33–99.73)	93.65 (84.16–104.2)	0.82 (0.62–1.09)	0.170
HPV33	80.00 (59.50–107.6)	91.76 (83.46–100.9)	0.87 (0.64–1.19)	0.387
HPV45	88.89 (67.94–116.3)	93.18 (82.75–104.9)	0.95 (0.71–1.28)	0.753
HPV52	90.00 (66.66–121.5)	88.89 (81.64–96.78)	1.01 (0.74–1.38)	0.938
HPV58	57.14 (43.79–74.57)	95.65 (89.03–102.8)	0.60 (0.45–0.79)	<0.001
HPV6	63.64 (47.68–84.93)	85.42 (78.51–92.93)	0.75 (0.55–1.01)	0.055
HPV11	84.62 (62.92–113.8)	86.86 (79.38–95.05)	0.97 (0.71–1.33)	0.868

HPV, human papillomavirus.

## Data Availability

De-identified participant data collected for the study, including individual participant data and a data dictionary defining each field in the set, will be made available from the corresponding author upon reasonable request.

## Abbreviations

The following abbreviations are used in this manuscript:

HPV	Human Papillomavirus
MSM	Men who have sex with men
NGO	Non-Governmental Organizations
PR	Prevalence ratio
IR	Incidence rate
CR	Clearance rate
IRR	Incidence rate ratio
IQR	Interquartile ranges

## References

[1] Biological Agents, IARC Working Group on the Evaluation of Carcinogenic Risks to Humans (2012). IARC Monographs on the Evaluation of Carcinogenic Risks to Humans.

[2] McBride A.A. (2022). Human papillomaviruses: Diversity, infection and host interactions. Nat. Rev. Microbiol..

[3] Doorbar J., Quint W., Banks L., Bravo I.G., Stoler M., Broker T.R., Stanley M.A. (2012). The biology and life-cycle of human papillomaviruses. Vaccine.

[4] de Martel C., Plummer M., Vignat J., Franceschi S. (2017). Worldwide burden of cancer attributable to HPV by site, country and HPV type. Int. J. Cancer.

[5] Hotca A., Goodman K.A. (2023). Trends in Anal Cancer: Leveraging Public Health Efforts to Improve Cancer Care. J. Clin. Oncol..

[6] Malagón T., Franco E.L., Tejada R., Vaccarella S. (2024). Epidemiology of HPV-associated cancers past, present and future: Towards prevention and elimination. Nat. Rev. Clin. Oncol..

[7] Clifford G.M., Georges D., Shiels M.S., Engels E.A., Albuquerque A., Poynten I.M., de Pokomandy A., Easson A.M., Stier E.A. (2021). A meta-analysis of anal cancer incidence by risk group: Toward a unified anal cancer risk scale. Int. J. Cancer.

[8] Dreyer G. (2018). Clinical implications of the interaction between HPV and HIV infections. Best Pract. Res. Clin. Obstet. Gynaecol..

[9] Tian T., Mijiti P., Bingxue H., Fadong Z., Ainiwaer A., Guoyao S., Zhanlin Z., Mahan Y., Xiaoqin T., Zheng G. (2017). Prevalence and risk factors of anal human papillomavirus infection among HIV-negative men who have sex with men in Urumqi city of Xinjiang Uyghur Autonomous Region, China. PLoS ONE.

[10] Kasavandi A., Foroohi F., Rahimi T., Ferdousi A., Mohammadian T. (2024). An Insight into the Coinfections of Hepatitis B, C, and Mycobacterium Tuberculosis in Iranian HIV Patients. Iran. Red Crescent Med. J. (IRCMJ).

[11] Hargreaves J.R., Bonell C.P., Boler T., Boccia D., Birdthistle I., Fletcher A., Pronyk P.M., Glynn J.R. (2008). Systematic review exploring time trends in the association between educational attainment and risk of HIV infection in sub-Saharan Africa. AIDS.

[12] Hu H., Hao J., Wang D., Liu X., Chen H., Li F., Chen J., Li M., Xin P., Li Y. (2025). Pretreatment HIV Drug Resistance to Integrase Strand Transfer Inhibitors Among Newly Diagnosed HIV Individuals—China, 2018–2023. China CDC Wkly..

[13] Shan D., Liu Y., Zang C., Zhao Y., Li H., Han J., Yang J., Liu J., Liu Z., Liu Y. (2025). Characteristics and Predictors of Interprovincial Migration Following HIV Diagnosis Among Men Who Have Sex with Men—China, 2016–2022. China CDC Wkly..

[14] Sandfort T.G., Nel J., Rich E., Reddy V., Yi H. (2008). HIV testing and self-reported HIV status in South African men who have sex with men: Results from a community-based survey. Sex. Transm. Infect..

[15] Outlaw A.Y., Phillips G., Hightow-Weidman L.B., Fields S.D., Hidalgo J., Halpern-Felsher B., Green-Jones M. (2011). Age of MSM sexual debut and risk factors: Results from a multisite study of racial/ethnic minority YMSM living with HIV. AIDS Patient Care STDS.

[16] Brown M.J., Serovich J.M., Laschober T.C., Kimberly J.A. (2018). Age and racial disparities in substance use and self-reported viral suppression among men who have sex with men with HIV. Int. J. STD AIDS.

[17] Pankam T., Kerr S.J., Teeratakulpisan N., Rodbamrung P., Wongkanya R., Keelawat S., Ruangritchankul K., Hongchookiat P., Watanapokasin R., Phanuphak N. (2017). Human papillomavirus in anal biopsy tissues and liquid-based cytology samples of HIV-positive and HIV-negative Thai men who have sex with men. Papillomavirus Res..

[18] Zhang D.Y., Yin Y.P., Feng T.J., Hong F.C., Jiang N., Wang B.X., Chen X.S. (2014). HPV infections among MSM in Shenzhen, China. PLoS ONE.

[19] Li X., Li M., Yang Y., Zhong X., Feng B., Xin H., Li Z., Jin Q., Gao L. (2016). Anal HPV/HIV co-infection among Men Who Have Sex with Men: A cross-sectional survey from three cities in China. Sci. Rep..

[20] Donà M.G., Giuliani M., Rollo F., Vescio M.F., Benevolo M., Giglio A., Giuliani E., Morrone A., Latini A. (2022). Incidence and clearance of anal high-risk Human Papillomavirus infection and their risk factors in men who have sex with men living with HIV. Sci. Rep..

[21] Wei F., Gaisa M.M., D’Souza G., Xia N., Giuliano A.R., Hawes S.E., Gao L., Cheng S.H., Donà M.G., Goldstone S.E. (2021). Epidemiology of anal human papillomavirus infection and high-grade squamous intraepithelial lesions in 29,900 men according to HIV status, sexuality, and age: A collaborative pooled analysis of 64 studies. Lancet HIV.

[22] Ucciferri C., Tamburro M., Falasca K., Sammarco M.L., Ripabelli G., Vecchiet J. (2018). Prevalence of anal, oral, penile and urethral Human Papillomavirus in HIV infected and HIV uninfected men who have sex with men. J. Med. Virol..

[23] Geskus R.B., González C., Torres M., Del Romero J., Viciana P., Masiá M., Blanco J.R., Iribarren M., De Sanjosé S., Hernández-Novoa B. (2016). Incidence and clearance of anal high-risk human papillomavirus in HIV-positive men who have sex with men: Estimates and risk factors. AIDS.

[24] Wei F., Goodman M.T., Xia N., Zhang J., Giuliano A.R., D’Souza G., Hessol N.A., Schim van der Loeff M.F., Dai J., Neukam K. (2023). Incidence and Clearance of Anal Human Papillomavirus Infection in 16 164 Individuals, According to Human Immunodeficiency Virus Status, Sex, and Male Sexuality: An International Pooled Analysis of 34 Longitudinal Studies. Clin. Infect. Dis..

